# Meteorological Observations

**Published:** 1859-11

**Authors:** J. G. Westmoreland

**Affiliations:** Smithsonian Institution, Oct., 1859, at Atlanta, Ga.


### Meteorological Observations,

**Taken by J. G. Westmoreland, M. D., for the Smithsonian Institution, Oct., 1859, at Atlanta, Ga., Latitude 33 deg. 45 min--Longitude 7 deg. 30 min.--Height above the Sea, 1050 feet.**

| Days of Mo. | BAROMETER. | | | THERMOMETER. | | | RAIN. | CLOUDS. | | | WIND. | | |
|---|---|---|---|---|---|---|---|---|---|---|---|---|---|
| | 7 A. M. | 2 P. M. | 9 P. M. | 7 A. M. | 2 P. M. | 8 P. M. | | 7 A.M. | 2 P.M. | 9 P.M. | 7 A.M. | 2 P.M. | 9 P.M. |
| 1 | 30.15 | 30.12 | 30.10 | 64 | 76 | 68 | | 5 | 5 | 10 | E | E | E |
| 2 | 30.18 | 30.10 | 30.10 | 58 | 68 | 60 | | 10 | 5 | 0 | E | E | W |
| 3 | 30.10 | 30.15 | 30.12 | 54 | 72 | 64 | 37 | 0 | 0 | 0 | N W | W | W |
| 4 | 30.05 | 30.10 | 30.05 | 56 | 78 | 66 | | 0 | 0 | 0 | W | W | W |
| 5 | 30.05 | 30.10 | 30.05 | 54 | 74 | 60 | | 0 | 0 | 0 | W | W | W |
| 6 | 30.05 | 30.15 | 30.05 | 56 | 74 | 60 | | 0 | 0 | 0 | W | W | W |
| 7 | 30.05 | 30.10 | 30.05 | 54 | 74 | 62 | | 5 | 5 | 5 | S W | W | W |
| 8 | 29.90 | 29.85 | 29.85 | 60 | 78 | 60 | 25 | 5 | 10 | 10 | E | S W | W |
| 9 | 29.85 | 29.95 | 29.95 | 46 | 68 | 50 | | 5 | 5 | 0 | N W | N W | N W |
| 10 | 30.00 | 30.10 | 30.10 | 40 | 62 | 56 | | 0 | 0 | 0 | N W | W | W |
| 11 | 30.10 | 30.20 | 30.15 | 48 | 70 | 60 | | 0 | 0 | 0 | W | W | W |
| 12 | 30.15 | 30.20 | 30.15 | 50 | 68 | 60 | | 0 | 5 | 5 | E | E | E |
| 13 | 30.12 | 30.15 | 30.10 | 55 | 70 | 65 | | 10 | 5 | 5 | W | S E | S E |
| 14 | 30.10 | 30.12 | 30.05 | 60 | 76 | 66 | | 5 | 5 | 5 | S E | S E | S E |
| 15 | 30.05 | 30.12 | 30.15 | 58 | 74 | 58 | | 5 | 0 | 0 | S E | S E | E |
| 16 | 30.15 | 30.18 | 30.15 | 50 | 64 | 60 | 25 | 5 | 5 | 5 | E | E | E |
| 17 | 30.15 | 30.10 | 30.08 | 62 | 68 | 56 | 75 | 10 | 10 | 10 | E | E | E |
| 18 | 30.00 | 30.00 | 29.95 | 60 | 62 | 50 | | 10 | 5 | 0 | S E | N W | N W |
| 19 | 29.90 | 29.90 | 29.85 | 40 | 60 | 50 | | 0 | 0 | 0 | N W | N W | N W |
| 20 | 29.85 | 29.92 | 29.95 | 42 | 64 | 50 | | 0 | 0 | 0 | N W | N W | N W |
| 21 | 30.00 | 30.00 | 29.95 | 44 | 60 | 46 | | 0 | 0 | 0 | E | E | E |
| 22 | 29.90 | 30.00 | 29.95 | 42 | 64 | 54 | | 0 | 0 | 0 | E | E | E |
| 23 | 30.00 | 30.15 | 30.12 | 48 | 70 | 56 | | 0 | 0 | 0 | S E | S E | S E |
| 24 | 30.10 | 30.05 | 30.05 | 48 | 70 | 62 | | 0 | 0 | 0 | S E | S E | S E |
| 25 | 30.00 | 30.00 | 29.90 | 56 | 78 | 62 | | 0 | 0 | 0 | N W | N W | W |
| 26 | 29.80 | 29.80 | 29.68 | 56 | 80 | 60 | | 0 | 0 | 0 | W | W | W |
| 27 | 29.65 | 29.70 | 29.70 | 50 | 70 | 50 | | 0 | 0 | 0 | W | W | W |
| 28 | 29.70 | 29.63 | 29.80 | 36 | 56 | 42 | | 0 | 0 | 0 | N W | N W | N W |
| 29 | 29.90 | 30.00 | 30.00 | 34 | 50 | 36 | | 0 | 0 | 0 | N W | N W | N W |
| 30 | 30.10 | 30.05 | 30.05 | 34 | 50 | 42 | | 0 | 0 | 0 | N W | N W | N W |
| 31 | 29.95 | 30.00 | 30.00 | 34 | 52 | 44 | | 0 | 0 | 0 | N W | N W | N W |

EXPLANATION OF TABLE.—0 Signifies Entire Clearness—5 Half Cloudy—10 Entire Cloudiness.

